# The “bubbles”-study: Validation of ultra-short scales for the assessment of depression, anxiety and stress symptoms

**DOI:** 10.1371/journal.pone.0300923

**Published:** 2024-03-20

**Authors:** Julia Brailovskaia, Silvia Schneider, Jürgen Margraf

**Affiliations:** 1 Mental Health Research and Treatment Center, Department of Clinical Psychology and Psychotherapy, Ruhr-Universität Bochum, Bochum, Germany; 2 DZPG (German Center for Mental Health), Partner Site Bochum/Marburg, Germany; 3 Mental Health Research and Treatment Center, Department of Clinical Child and Adolescent Psychology, Ruhr-Universität Bochum, Bochum, Germany; Walailak University, THAILAND

## Abstract

Depression, anxiety and stress symptoms cause substantial psychological and economic burdens around the globe. To mitigate the negative consequences, the negative symptoms should be identified at an early stage. Therefore, the implementation of very brief valid screening tools in mental health prevention programs and in therapeutic settings is advantageous. In two studies on representative German population samples, we developed and validated three ultra-short scales–the “bubbles”–that consist of only one item based on the Depression Anxiety Stress 21 subscales (DASS-21) for the assessment of depression, anxiety and stress symptoms. The results of Study 1 (*N* = 1,001) and Study 2 (*N* = 894) revealed that the bubbles are valid instruments that fit the DASS-21 subscales on the factor level. Moreover, the bubbles replicated the association pattern of the DASS-21 subscales with demographic variables, and with variables that belong to the negative and the positive dimension of mental health. Thus, due to their time- and cost-efficiency, the bubbles can be used as brief screening tools in research (e.g., large-scale studies, longitudinal studies, experience sampling paradigms) and in praxis. Their shortness can prevent fatigue, motivation decrease, and participants’ drop-out.

## Introduction

One of every eight people in the world suffers from a mental health problem [1]. Mental health problems have a substantial negative effect on all areas of life [2]. They restrict a person’s quality of life and cause high economic burden and substantial financial costs to the community through direct therapy costs and indirect costs caused by, for example, absence from workplace and mortality [3–5]. Depression but also anxiety and stress symptoms belong to the leading causes of disability worldwide [2]. Research from various countries reported a significant increase of the negative symptoms since the global outbreak of the coronavirus disease 2019 (Covid-19) [6–10]. The enhanced level of depression, anxiety, and stress symptoms has been accompanied by a significant increase of suicide-related outcomes (i.e., suicide ideation and suicide attempts) in the general population and in inpatients [11–13].

Against this background, it is of great importance to identify the level of the negative symptoms at an early stage to prevent their further development and maintenance. To achieve this, we need very brief valid screening tools that can be implemented in mental health prevention programs and in therapeutic settings [14]. Currently there is a lack of such very brief validated screening tools. This often results in high drop-out rates especially in time-restricted conditions like large longitudinal panel surveys and in vulnerable populations such as clinical patients with a low attention span who cannot concentrate on long questionnaires [15, 16]. Thus, the development and validations of such brief tools is strongly desirable [17].

In the clinical context, the Beck Depression Inventory (BDI) [18, 19] and the Beck Anxiety Inventory (BAI) [20] are commonly used instruments for the assessment of depressiveness and anxiety. Both instruments consist of, respectively, 21 items which is long for a rapid general screening. Lovibond and Lovibond (21) demonstrated the validity of the Depression Anxiety Stress Scales 21 (DASS-21) that assess symptoms of depression, anxiety and stress over the past week with respectively seven items per subscale. The DASS-21 is closely linked to the Beck’s Inventories [21]. Its psychometric properties and measurement invariance have been shown across various countries [22–26]. Today, the DASS-21 belongs to the internationally well-established and well-validated instruments for the assessed of the negative symptoms in general population and in clinical setting [27–30]. However, 21 items seem still to be not brief enough for a rapid mental health screening. Shorter scales–especially single-items scales–would be of advantage [14, 25, 26, 31].

Previous research reported some shortcomings of single-item scales such as simplifying multidimensional topics and not being able to assess fine-grained differences between individuals [32]. However, they also have remarkable advantageous. The single-item scales are extremally time-saving that prevents fatigue, a decrease of motivation and participants’ drop-out [33]. Therefore, they can be used for brief screenings in clinical settings where patients often display a low attention span. Moreover, large representative studies and longitudinal studies with several measurement time points can benefit from the shortness of such time- and cost-efficient instruments. Available literature described single-items scales that assess constructs such as self-esteem [34], risk-taking [35], narcissism [33], need to belong [36], Fear of Missing Out [37], and happiness [38] to have adequate psychometric properties and to show similar valid results as the long-version measures of the constructs.

Against this background, we aimed to develop and validate ultra-short scales that consist of only one item based on the DASS-21 subscales for the assessment of depression, anxiety and stress symptoms. Therefore, we conducted two studies in population representative samples in Germany as part of a large ongoing project that investigates mental health factors. Notably, the second study aimed mainly to replicate the findings of the first study. The replication is important considering the often described replication crisis in psychology research [39].

In Study 1, we developed three ultra-short scales termed as “depression bubble”, “anxiety bubble”, and “stress bubble” based on expert reviews. Then, we investigated the validity of the bubbles. Therefore, we assessed whether each bubble belongs to the same unidimensional factor as the seven items of the corresponding DASS-21 subscale, we focused on the relationship between the bubbles and the DASS-21 subscales, and we compared their association pattern with other variables that previous research described to be associated with depression, anxiety and stress symptoms.

Considering the association pattern, we focused on available literature. In addition to the association between the bubbles and the symptoms of depression, anxiety and stress as assessed by the DASS-21, we involved demographic variables (age, gender, social status) and the positive mental health (PMH) in the analyses. It is worth noting that previous research has reported inconclusive findings regarding the relationship between negative symptoms and age. Some studies described a positive association [40], other reported a negative link [41–43]. Schönfeld, Brailovskaia [44] found a positive association between age and the negative symptoms in Russia, a negative association in Germany and no significant association in the U.S. Considering gender, higher levels of depression, anxiety and stress symptoms were reported for women than for men [45–49]. Furthermore, the negative symptoms were negatively associated with social status [50–52].

Notably, mental health is not only the absence of psychopathology [2]. Following dual-factor models, mental health consists of two distinct but correlated dimensions: negative and positive [53]. A status of absolute mental health is characterized by a low level of the negative dimension and a high level of the positive dimension [54]. The negative dimension is often operationalized by depression, anxiety and stress symptoms, the positive dimension can be operationalized by positive mental health (PMH) [55, 56]. There is a close positive relationship between the negative symptoms [21, 57]. PMH, i.e., emotional, cognitive and psychological well-being, is negatively associated with the negative symptoms as assessed by the DASS-21 subscales [58–60]. Thus, it was important to assess whether the bubbles are also negatively associated with the positive dimension of mental health operationalized by PMH. Findings on this issue would provide evidence of whether the bubbles show a similar association pattern as the DASS-21, considering the overall mental health construct, including its both dimensions, and therefore contribute to our understanding of the bubbles’ validity (specifically its convergent validity).

Study 2 replicated and extended the findings of Study 1 on the validity of the bubbles. After a further investigation of the question, whether each bubble belongs to the same unidimensional factor as the seven items of the corresponding DASS-21 subscale, we focused again on the associations of the bubbles in comparison to the DASS-21 subscales. Hereby, we extended the examination of the convergent validity of the bubbles by the inclusion of sense of control (negatively coded) that is a further operationalization of the negative dimension of mental health [61] and the inclusion of life satisfaction and social support that are often used in addition to PMH to operationalize the positive dimension of mental health [62]. Previous research has described that a low level of sense of control contributes to higher levels of depression, anxiety and stress symptoms [7, 60, 63]; the negative symptoms were negatively associated with life satisfaction [56, 64, 65], and persons who perceived a high level of social support from their close social network were at a lower risk for the negative symptoms [43, 66]. Thus, the inclusion of the three new variables should extend our understanding of the associations of the bubbles with further representatives of both mental health dimensions and their (convergent) validity. A similar association pattern to that of the DASS-21 could provide further evidence for the suitability of using the bubbles as a replacement for the DASS-21 scales in for example longitudinal research.

Overall, after the development of the bubbles, the findings of both studies should provide initial evidence for their validity.

## General methods

For both studies, data were collected in June 2023 by an independent social marketing and research institute (Study 1: Talk Online, www.talkonlinepanel.com/de; Study 2: YouGov, www.yougov.de) via a population-based online-panel survey. Participants were recruited from the German residential population aged 18 years and above. Age (Talk Online: 18 to 29 years, 30 to 39 years, 40 to 49 years, 50 to 59 years, 60 years and older; YouGov: 18 to 24 years, 25 to 34 years, 35 to 44 years, 45 to 54 years, 55 years and older), gender (Talk Online and YouGov: male persons, female persons, gender diverse persons), and region/federal state (Talk Online and YouGov: the 16 German federal states) stratification were implemented to achieve representativeness for the German population. Notably, both institutes have a large number of panel-members from the residential population. The institutes invite members of their panel to participate in the survey as long as the demographics of the present sample correspond to those of the German population in terms of age, gender and region/federal state rates.

Participation was compensated by panel-specific tokens that can be converted in voucher or monetary payment. The requirement for participation was legal age (at least 18 years). There were no specific inclusion or exclusion criteria other than to belong to the residential population aged 18 years and above in Germany. All participants were properly instructed and gave online their informed consent to participate. The survey data were not analyzed at the level of individual persons. Participants were informed that it is not possible to clarify any conspicuous findings on mental health variables, as the data obtained are only evaluated in aggregated and anonymized form (no identification of individual persons is possible). The responsible Ethics Committee approved the current study’s implementation (application number: 20110512). It was pre-registered with AsPredicted.org on May 03, 2023 (Pre-registration Number: #130926). All national regulations and laws regarding human subjects research were followed. The study was conducted in accordance with the Declaration of Helsinki. All data sets used in the present study were complete. Following available literature, the calculation of a confirmatory factor analyses (CFA)–that was a significant part of Study 2 –requires at least a sample size of *N* = 200 for valid results [67]; considering an exploratory factor analyses (EFA) that was a significant part of Study 1, there are various suggestions on the required sample size that vary between *N* = 100 and 500 [67]. A priori calculated power analyses (G*Power program, version 3.1) showed that the correlation and regression analyses that were also calculated in the present study required a sample size below *N* = 200 [68]. However, considering that we aimed to develop new instruments, we decided to collect data of *N* = 800 to 1000 in each study.

## Study 1

### Methods

#### Procedure and participants

Overall, 1,163 started the survey and 162 (13.9%) dropped out. Thus, the sample of Study 1 included 1,001 participants from Germany. Table 1 shows the demographic variables derived from the present sample. The dataset used in the present study is available in S1 Dataset.

**Table 1:** Demographic variables derived from the present samples of Study 1 and Study 2.

	Study 1 (*N* = 1,001)	Study 2 (*N* = 894)
*Age (years)*		
*M* (*SD*; Min–Max)	49.36 (17.36; 18–93)	51.23 (16.05; 18–89)
*Gender (%)*		
Women	50.7	54.7
Men	49.3	45.3
*Marital Status (%)*		
Single	26.3	25.7
Romantic relationship, not married	13.5	13.8
Married	44.5	43.3
Widowed, divorced	15.7	17.2
*Occupation* (%)		
Student	7.5	4.3
Employee	60.7	63.0
Unemployment	5.3	4.6
Retire	26.5	28.1
*Living Environment (%)*		
Large city	37.7	35.0
Small city	39.3	39.1
Rural community	23.0	25.9
*Social Status (%)*		
Lower class	8.4	7.4
Working class	17.5	17.4
Lower middle class	24.0	25.5
Middle middle class	40.0	37.7
Upper middle class	9.0	11.3
Upper class	1.1	0.7

*Notes*. Study 1 and Study 2: There were no “gender divers” participants.

#### Measures

*Demographics*. Participants were asked to indicate their gender, age, marital status, occupation, living environment, and social status.

*Depression*, *anxiety*, *and stress symptoms*. The Depression Anxiety Stress Scales 21 (DASS-21; original version: [21]; German language version: [69]) assessed symptoms of depression, anxiety and stress over the past week with respectively seven items per subscale (e.g., depression subscale: “I felt that life was meaningless”; anxiety subscale: “I felt scared without any good reason”; stress subscale: “I found it hard to wind down”). Previous research described the unidimensional factor structure of the three DASS-21 subscales [21]. The 21 items are rated on a 4-point Likert-type scale (0 = *did not apply to me at all*, 3 = *applies to me very much or most of the time*). Higher sum scores indicate higher symptoms. The total sum score of each subscale can range from zero to 21. Current scale reliability was *α* = .936 for the depression subscale, *α* = .914 for the anxiety subscale, and *α* = .917 for the stress subscale.

**Fig 1:**
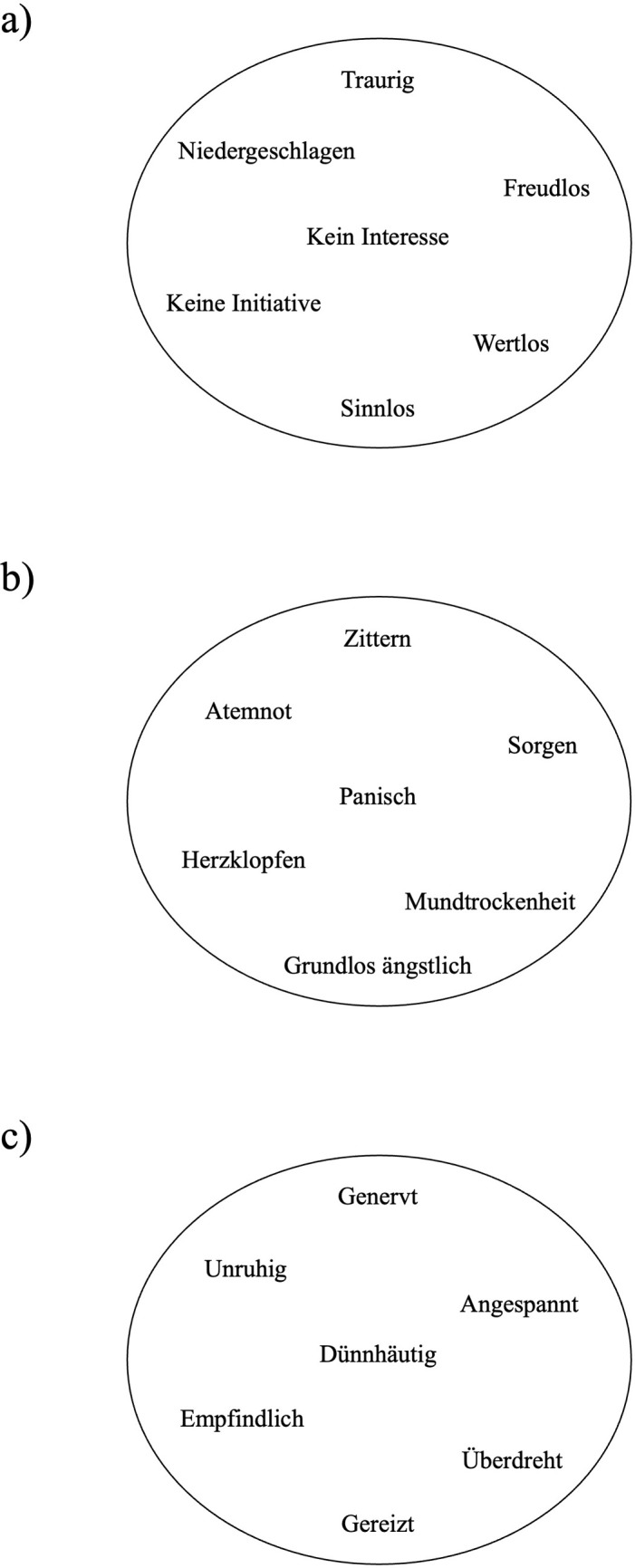
Original German language Bubbles: a) Depression Bubble; b) Anxiety Bubble; c) Stress Bubble.

*Bubbles*: *Depression*, *anxiety and stress*. We developed three bubbles–one per subscale of the DASS-21 –based on the items of the DASS-21 [69]. By the implementation of expert reviews by two psychology trained professionals, who evaluated the appropriateness of context, conciseness and wording, each of the seven items of a subscale was shortened to one word or a brief phrase consisting of several words. For example, the item of the DASS-21 depression subscale “I felt that life was meaningless” was shortened to the word “meaningless”; the item of the DASS-21 anxiety subscale “I felt scared without any good reason” was shortened to the phrase “groundless scared”; and the item of the DASS-21 stress subscale “I found it hard to wind down” was shortened to the word “restless”. Thus, the exact wording of the depression bubble was “sad”, “no initiative”, “joyless”, “depressed”, “no interest”, “worthless”, “meaningless”; the exact wording of the anxiety bubble was “dryness of mouth”, “breathlessness”, “tremble”, “worries”, “panic”, “beating of the heart”, “groundless scared”; and the exact wording of the stress bubble was “restless”, “irritated”, “tense”, “agitated”, “overexcited”, “thin-skinned”, “sensitive” (see Table 2 and Fig 1). The words/phrases that belong to a subscale were included in one visual bubble. Thus, each bubble can be considered as a single-item scale/instrument that includes the content of the seven items from the corresponding DASS-21 subscale in a shortened form. Participants had to rate how much/how often the overall content of each bubble applied to them over the past week (“Please rate how much/how often the overall content of the bubble applied to you over the past week”). Each bubble was rated on a horizontally arranged 4-point Likert-type scale (1 = *not at all*, 2 = *a little bit / sometimes*, 3 = *substantial / often*, 4 = *very strong / mostly*). The rating scale was presented below each bubble. The higher the rating, the higher the negative symptom represented by the bubble. For example, if a participant rated the depression bubble with “2” on the 4-point Likert-type scale, the person had a score of 2 for symptoms of depression.

**Table 2:** Content of the depression, anxiety and stress bubbles in relationship with the items of the Depression Anxiety Stress Scales 21.

Original Item	Exact wording of the Bubble
*DASS-21*: *Depression Subscale*	*Depression Bubble*
Item 1	sad
Item 2	no initiative
Item 3	joyless
Item 4	depressed
Item 5	no interest
Item 6	worthless
Item 7	meaningless
*DASS-21*: *Anxiety Subscale*	*Anxiety Bubble*
Item 1	dryness of mouth
Item 2	breathlessness
Item 3	tremble
Item 4	worries
Item 5	panic
Item 6	beating of heart
Item 7	groundless scared
*DASS-21*: *Stress Subscale*	*Stress Bubble*
Item 1	restless
Item 2	irritated
Item 3	tense
Item 4	agitated
Item 5	overexcited
Item 6	thin-skinned
Item 7	sensitive

*Notes*. DASS-21 = Depression Anxiety Stress Scales.

*Positive Mental Health (PMH)*. The unidimensional Positive Mental Health Scale (PMH-Scale; original German language version: [70]) measured the level of PMH. The PMH-Scale is a well-established instrument for the assessment of emotional, cognitive and psychological well-being. It consists of nine items that are rated on a 4-point Likert-type scale (e.g., “I enjoy my life”; 0 = *do not agree*, 3 = *agree*). The higher the sum score, the higher the level of PMH. The total sum score can range from zero to 27. Current scale reliability is *α* = .937.

#### Statistical analyses

Statistical analyses were conducted using the Statistical Package for the Social Sciences (SPSS) version 28 [71]. All investigated psychological variables were close to normally distributed (indicated by analyses of skewness, < 2.00, and kurtosis, < 7.00 [72]; see Table 4). We investigated the psychometric properties of the three bubbles. First, we calculated three EFAs to investigate whether each bubble belongs to the same unidimensional factor as the seven items of the corresponding DASS-21 subscale. Notably, available literature emphasized that depression, anxiety, and stress symptoms should be considered as separate entities [73, 74]. Often, a study focuses only one of those constructs [75]. Against this background, we made the decision to calculate an EFA for each construct separately instead of an EFA that includes all three constructs (that are 24 items: 21 DASS items and the three bubbles). Therefore, for each negative symptom, we ran an EFA for the seven items of the DASS-21 subscale with the corresponding bubble using the Maximum Likelihood method (ML; rotation method: promax) [76, 77]. Thus, each of the three EFAs included eight items (i.e., seven DASS-21 items and one bubble). Furthermore, we used the factor loadings of the EFAs to calculate the average variance extracted (AVE) for conclusions on the convergent validity of the bubbles. Convergent validity is established for AVE ≥ .50 [78].

In the next step, we assessed the reliability of the bubbles. As we were not able to investigate their test-retest reliability due to the cross-sectional study design, we calculated the internal consistency (Cronbach’s *α*) and the composite reliability (CR) [78] of the bubbles in relationship with the DASS-21 subscales to assess their reliability. This step revealed the internal consistency of the DASS-21 subscale items when adding the corresponding bubble. Considering that the bubbles aim to assess the same content as the DASS-21 subscale items, a high internal consistency provides support for the reaching of this aim.

Then, to further assess the validity of the bubbles and their closeness to the original scales, we investigated the correlation pattern of the bubbles with variables that were earlier shown to the linked to depression, anxiety and stress symptoms. Specifically, we assessed their association with demographic variables (age, gender, social status), the DASS-21 subscales and the PMH-Scale by the calculation of Pearson’s zero-order bivariate correlations and Spearman’s rank order correlations [79]. The associations with the mental health variables allowed further conclusions on the convergent validity of the bubbles. Furthermore, we compared the correlations of the bubbles with those of the corresponding DASS-21 subscale to test whether they exhibit a similar correlation pattern. This step revealed further information on the convergent validity of the bubbles. Following Cohen [80], we used the effect size Cohen’s q (small effect: .10 ≤ q < .30; medium effect: .30 ≤ q ≤ .50; large effect: q > .50) for the comparison of the correlations (bubble vs. DASS-21 subscale). To assess the potential predictive power of the bubbles in comparison to the power of the DASS-21 subscales, we calculated linear regression analyses that included, respectively, one of the bubbles as predictor and, respectively, the DASS-21 subscales and the PMH-Scale as outcome. Then, we replicated the regression analyses with the DASS-21 subscales, respectively, as predictor. The cross-sectional study design does not allow true conclusions on causality. However, the findings allowed a further comparison of both measures.

### Results

#### Factor structure of the bubbles in relationship with the DASS-21 subscales: Exploratory Factor Analyses (EFAs)

*Depression symptoms*. The first EFA that included the DASS-21 depression subscale and the depression bubble (Kaiser-Meyer-Olkin: KMO = .951; Barlett’s test of sphericity: *χ*^*2*^ = 5864.526, df = 28, *p* < .001) revealed a unidimensional factor structure. The eigenvalue of the factor was 5.204, and it explained 65.1% of the variance which is sufficient for a one-factor structure [81]. Table 3 shows the factor loadings of the 8 items. The AVE was .650 revealing an adequate convergent validity.

**Table 3:** Factor loadings of the exploratory factor analyses (Depression Anxiety Stress Scales 21 and bubbles; Study 1).

Exploratory Factor Analyses	Factor Loading of Factor 1
*1*. *EFA*: *Depression Symptoms*	
DASS-21: Depression Subscale Item 1	.832
DASS-21: Depression Subscale Item 2	.735
DASS-21: Depression Subscale Item 3	.854
DASS-21: Depression Subscale Item 4	.845
DASS-21: Depression Subscale Item 5	.833
DASS-21: Depression Subscale Item 6	.827
DASS-21: Depression Subscale Item 7	.832
Depression Bubble	.675
*2*. *EFA*: *Anxiety Symptoms*	
DASS-21: Anxiety Subscale Item 1	.687
DASS-21: Anxiety Subscale Item 2	.756
DASS-21: Anxiety Subscale Item 3	.745
DASS-21: Anxiety Subscale Item 4	.788
DASS-21: Anxiety Subscale Item 5	.824
DASS-21: Anxiety Subscale Item 6	.828
DASS-21: Anxiety Subscale Item 7	.811
Anxiety Bubble	.650
*3*. *EFA*: *Stress Symptoms*	
DASS-21: Stress Subscale Item 1	.784
DASS-21: Stress Subscale Item 2	.711
DASS-21: Stress Subscale Item 3	.824
DASS-21: Stress Subscale Item 4	.806
DASS-21: Stress Subscale Item 5	.792
DASS-21: Stress Subscale Item 6	.762
DASS-21: Stress Subscale Item 7	.797
Stress Bubble	.585

*Notes*. *N* = 1,001; EFA = Exploratory Factor Analysis; DASS-21 = Depression Anxiety Stress Scales 21; overall three exploratory factor analyses were calculated, each of them included eight items (= seven items of the DASS-21 subscale and the corresponding bubble).

*Anxiety symptoms*. The findings for anxiety symptoms were similar to those of depression symptoms. The EFA that included the DASS-21 anxiety subscale and the anxiety bubble revealed a unidimensional factor structure (KMO = .940; Barlett’s test of sphericity: *χ*^*2*^ = 4710.614, df = 28, *p* < .001). The eigenvalue of the factor was 4.665 and it explained 58.3% of the variance (see Table 3 for factor loadings). The AVE was .583 revealing an adequate convergent validity.

*Stress symptoms*. Also, the factor analysis that included the DASS-21 stress subscale and the stress bubble revealed a unidimensional factor structure (KMO = .935; Barlett’s test of sphericity: *χ*^*2*^ = 4710.726, df = 28, *p* < .001). The eigenvalue of the factor was 4.635 and it explained 57.9% of the variance (see Table 3 for factor loadings). The AVE was .579 revealing an adequate convergent validity.

#### Reliability of the bubbles in relationship with the DASS-21 subscales

The overall scale reliability of the seven items of the DASS-21 depression subscale and the depression bubble was *α* = .935, and their composite reliability was .937; for the seven items of the DASS-21 anxiety subscale and the anxiety bubble, the overall scale reliability was *α* = .916, and the CR was .917; for the seven items of the DASS-21 stress subscale and the stress bubble, the overall scale reliability was *α* = .916, and the CR was .916.

#### Association pattern of the bubbles and convergent validity

The descriptive statistics (that are means, standard deviations, minimum, maximum) of the bubbles and the other assessed variables are presented in Table 4. Table 5 displays the correlations between the investigated variables. Notably, the bubbles and the corresponding DASS-21 subscales showed very similar correlation pattern.

**Table 4:** Descriptive statistics and properties of the investigated variables (Study 1, Study 2).

	*Study 1*, *N = 1*,*001*					*Study 2*, *N = 894*		
	*M(SD)*	*Min–Max*	*Skew*	*Kurt*	*M(SD)*	*Min–Max*	*Skew*	*Kurt*
DASS-21: Depression Subscale	5.17(5.30)	0–21	.976	.128	5.38(5.23)	0–21	.954	.092
Depression Bubble	1.99(.94)	1–4	.653	-.471	1.90(.88)	1–4	.749	-.173
DASS-21: Anxiety Subscale	4.27(4.87)	0–21	1.148	.443	3.98(4.63)	0–21	1.390	1.398
Anxiety Bubble	1.71(.90)	1–4	1.048	.067	1.53(.75)	1–4	1.308	1.049
DASS-21: Stress Subscale	5.60(5.00)	0–21	.721	-.311	6.41(5.04)	0–21	.636	-.210
Stress Bubble	2.16(.81)	1–4	.379	-.253	2.21(.86)	1–4	.392	-.445
Sense of Control					2.45(2.20)	0–8	.707	-.281
PMH-Scale	16.94(6.43)	0–27	-.626	.133	16.85(6.28)	0–27	-.364	-.354
SWLS					22.19(7.16)	5–35	-.369	-.463
F-SozU K-6					21.72(6.12)	6–30	-.607	-.284

*Notes*. *M* = Mean; *SD* = Standard Deviation; *Min* = Minimum; *Max* = Maximum; *Skew* = Skewness; *Kurt* = Kurtosis; DASS-21 = Depression Anxiety Stress Scales 21; PMH-Scale = Positive Mental Health Scale; SWLS = Satisfaction with Life Scale; F-Soz-U K-6 = Social Support Questionnaire.

**Table 5:** Correlations of the bubbles and Depression Anxiety Stress Scales 21 (Study 1, Study 2).

	Depression Bubble	DASS-21: Depression	Anxiety Bubble	DASS-21: Anxiety	Stress Bubble	DASS-21: Stress
*Study 1*, *N = 1*,*001*						
Age	-.331**	-.302**	-.259**	-.328**	-.348**	-.335**
Gender	.002	.005	.001	.001	-.065*	-.046
Social Status	-.149**	-.176**	-.098*	-.113**	-.097*	-.118**
DASS-21: Depression	.654**	1.000**	.561**	.817**	.507**	.862**
DASS-21: Anxiety	.554**	.817**	.621**	1.000**	.448**	.843**
DASS-21: Stress	.583**	.862**	.549**	.843**	.562**	1.000**
PMH-Scale	-.405**	-.469**	-.269**	-.298**	-.316**	-.395**
*Study 2*, *N = 894*						
Age	-.231**	-.260**	-.163**	-.234**	-.191**	-.282**
Gender	-.021	.010	.004	.013	-.084*	-.055
Social Status	-.268**	-.297**	-.190**	-.224**	-.191**	-.209**
DASS-21: Depression	.649**	1.000**	.599**	.810**	.502**	.842**
DASS-21: Anxiety	.480**	.810**	.680**	1.000**	.373**	.790**
DASS-21: Stress	.572**	.842**	.578**	.790**	.609**	1.000**
Sense of Control	.482**	.652**	.430**	.579**	.397**	.648**
PMH-Scale	-.520**	-.550**	-.344**	-.345**	-.455**	-.497**
SWLS	-.442**	-.417**	-.231**	-.217**	-.368**	-.343**
F-SozU K-6	-.345**	-.349**	-.204**	-.204**	-.224**	-.237**

*Notes*. Gender: coding 1 = women, 2 = men, point-biserial correlation; social status: Spearman’s rank order correlation; DASS-21 = Depression Anxiety Stress Scales 21; PMH-Scale = Positive Mental Health Scale; SWLS = Satisfaction with Life Scale; F-Soz-U K-6 = Social Support Questionnaire; Sense of Control: the higher the score, the lower the sense of control

**p < .001

*p < .05.

*Correlations with demographic variables*. As shown in Table 5, the three bubbles and the DASS-21 subscales were significantly negatively correlated with age. The correlations of the bubbles and the subscales did not show significant differences (bubble vs. DASS-21 subscale: depression: effect size q = .032, anxiety: q = .076, stress: q = .015; all: no effect).

The stress bubble was significantly negatively correlated with gender. The other bubbles and subscales were not significantly correlated with gender (see Table 5). There were no significant differences between the correlations of the bubbles and the subscales (bubble vs. DASS-21 subscale: depression: q = .076, anxiety: q = .0, stress: q = .019; all: no effect).

The three bubbles and the DASS-21 subscales were significantly negatively correlated with social status (see Table 5). The correlations did not significantly differ between the bubbles and the subscales (bubble vs. DASS-21 subscale: depression: q = .023, anxiety: q = .015, stress: q = .021; all: no effect).

*Convergent validity*. There was a significant positive correlation between depression, anxiety and stress symptoms–assessed by the bubbles and by the DASS-21 subscales. The depression bubble was significantly positively correlated with the anxiety bubble, *r* = .640, *p* < .001, and the stress bubble, *r* = .617, *p* < .001. The anxiety bubble was significantly positively correlated with the stress bubble, *r* = .549, *p* < .001. The positive association between the bubbles corresponds with the close positive relationship between the DASS-21 subscales (see Table 5).

Furthermore, Table 5 shows that the depression bubble was significantly positively correlated with the DASS-21 depression subscale. Both measures were significantly positively correlated with DASS-21 anxiety symptoms (DASS-21 > bubble: q = .524, large effect) and DASS-21 stress symptoms (DASS-21 > bubble: q = .634, large effect) (see Table 5). The anxiety bubble was significantly positively correlated with the DASS-21 anxiety subscale. Both measures were significantly positively correlated with DASS-21 depression symptoms (DASS-21 > bubble: q = .513, large effect) and DASS-21 stress symptoms (DASS-21 > bubble: q = .615, large effect). As shown in Table 5, the stress bubble was significantly positively correlated with the DASS-21 stress subscale. Both measures were significantly positively correlated with DASS-21 depression symptoms (DASS-21 > bubble: q = .742, large effect) and DASS-21 anxiety symptoms (DASS-21 > bubble: q = .749, large effect).

Moreover, the bubbles and the DASS-21 subscales were significantly negatively correlated with the PMH-Scale (see Table 5). The results of the bubbles did not significantly differ from those of the subscales (bubble vs. DASS-21 subscale: depression: q = .079, anxiety: q = .032, stress: q = .091; all: no effect).

#### Predictive power of the bubbles

Table 6 shows the results of the regression analyses. All investigated regression models were significant revealing that the bubbles could predict symptoms of depression, anxiety, and stress as well as PMH. Again, the association of the bubbles with the DASS-21 subscales was weaker than the association between the subscales. However, the association between the bubbles and the PMH-Scale did not remarkably differ from the association of the subscales and the PMH-Scale (see Table 6).

**Table 6:** Linear regression analyses (Study 1, Study 2).

	*Study 1*, *N = 1*,*001*			*Study 2*, *N = 894*		
	ß	95% CI	Adjusted *R*^*2*^	ß	95% CI	Adjusted *R*^*2*^
*Outcome*: *DASS-21*: *Depression Subscale*						
Model 1: Predictor → Depression Bubble	.654**	[3.440,3.972]	.472	.649**	[3.556,4.149]	.421
Model 2: Predictor → Anxiety Bubble	.561**	[2.993,3.596]	.315	.599**	[3.798,4.530]	.358
Model 3: Predictor → Stress Bubble	.507**	[2.985,3.689]	.257	.502**	[2.688,3.375]	.252
Model 4: Predictor → DASS-21: Anxiety Subscale	.817**	[.850,.928]	.668	.810**	[.872,.959]	.656
Model 5: Predictor → DASS-21: Stress Subscale	.862**	[.881,.948]	.744	.842**	[.837,.910]	.709
*Outcome*: *DASS-21*: *Anxiety Subscale*						
Model 1: Predictor → Depression Bubble	.554**	[2.615,3.154]	.307	.480**	[2.217,2.823]	.230
Model 2: Predictor → Anxiety Bubble	.817**	[.718,.784]	.386	.680**	[3.893,4.486]	.463
Model 3: Predictor → Stress Bubble	.448**	[2.373,3.044]	.201	.373**	[1.671,2.324]	.139
Model 4: Predictor → DASS-21: Depression Subscale	.621**	[3.090,3.615]	.668	.810**	[.683,.751]	.656
Model 5: Predictor → DASS-21: Stress Subscale	.843**	[.789,.854]	.710	.790**	[.688,.762]	.624
*Outcome*: *DASS-21*: *Stress Subscale*						
Model 1: Predictor → Depression Bubble	.583**	[2.859,3.388]	.340	.572**	[2.967,3.584]	.328
Model 2: Predictor → Anxiety Bubble	.549**	[2.751,3.325]	.301	.578**	[3.517,4.236]	.334
Model 3: Predictor → Stress Bubble	.562**	[3.164,3.801]	.315	.609**	[3.246,3.853]	.371
Model 4: Predictor → DASS-21: Depression Subscale	.862**	[.783,.843]	.744	.842**	[.778,.846]	.709
Model 5: Predictor → DASS-21: Anxiety Subscale	.843**	[.830,.899]	.710	.790**	[.817,.904]	.624
*Outcome*: *PMH-Scale*						
Model 1: Predictor → Depression Bubble	-.405**	[-3.178,-2.396]	.164	-.520**	[-4.109,-3.309]	.271
Model 2: Predictor → Anxiety Bubble	-.269**	[-2.341,-1.489]	.072	-.344**	[-3.392,-2.361]	.119
Model 3: Predictor → Stress Bubble	-.316**	[-2.992,-2.052]	.100	-.455**	[-3.723,-2.874]	.207
Model 4: Predictor → DASS-21: Depression Subscale	-.469**	[-.636,-.503]	.220	-.550**	[-.726,-.595]	.302
Model 5: Predictor → DASS-21: Anxiety Subscale	-.298**	[-.472,-.316]	.089	-.345**	[-.551,-.384]	.119
Model 6: Predictor → DASS-21: Stress Subscale	-.395**	[-.582,-.435]	.156	-.497**	[-.690,-.548]	.247
*Outcome*: *Sense of Control*						
Model 1: Predictor → Depression Bubble				.482**	[1.061,1.349]	.232
Model 2: Predictor → Anxiety Bubble				.430**	[1.087,1.435]	.185
Model 3: Predictor → Stress Bubble				.397**	[.857,1.165]	.158
Model 4: Predictor → DASS-21: Depression Subscale				.652**	[.254,.296]	.425
Model 5: Predictor → DASS-21: Anxiety Subscale				.579**	[.250,.301]	.335
Model 6: Predictor → DASS-21: Stress Subscale				.648**	[.261,.305]	.420
*Outcome*: *SWLS*						
Model 1: Predictor → Depression Bubble				-.442**	[-4.072,-3.115]	.196
Model 2: Predictor → Anxiety Bubble				-.231**	[-2.811,-1.594]	.054
Model 3: Predictor → Stress Bubble				-.368**	[-3.550,-2.540]	.136
Model 4: Predictor → DASS-21: Depression Subscale				-.417**	[-.653,-.489]	.174
Model 5: Predictor → DASS-21: Anxiety Subscale				-.217**	[-.435,-.237]	.042
Model 6: Predictor → DASS-21: Stress Subscale				-.343**	[-.575,-.399]	.118
*Outcome*: *F-SozU K-6*						
Model 1: Predictor → Depression Bubble				-.345**	[-2.823,-1.966]	.119
Model 2: Predictor → Anxiety Bubble				-.204**	[-2.184,-1.137]	.042
Model 3: Predictor → Stress Bubble				-.224**	[-2.036,-1.131]	.050
Model 4: Predictor → DASS-21: Depression Subscale				-.349**	[-.481,-.337]	.122
Model 5: Predictor → DASS-21: Anxiety Subscale				-.204**	[-.354,-.184]	.041
Model 6: Predictor → DASS-21: Stress Subscale				-.237**	[-.365,-.210]	.056

*Notes*. ß = standardized beta, CI = Confidence Interval

**p < .001; DASS-21 = Depression Anxiety Stress Scales 21; PMH-Scale = Positive Mental Health Scale; SWLS = Satisfaction with Life Scale; F-SozU K-6 = Social Support Questionnaire; each regression analysis includes one of the bubbles or one of the DASS-21 subscales as predictor (= each line including a predictor provides the results of a regression model).

### Discussion

The main aim of Study 1 was to develop three ultra-short scales, each consisting of one item, based on the subscales of the DASS-21 to assess the level of depression, anxiety, and stress symptoms. Therefore, the content of each DASS-21 item was shortened to single words or brief phrases. The words/phrases that belong to a DASS-21 subscale were included in a visual bubble (see Fig 1). In the next step, we provided initial findings regarding the validity of the three bubbles.

First, we calculated three EFAs, each containing eight items (seven items of a DASS-21 subscale and the corresponding bubble). Each EFA revealed a unidimensional factor structure. This indicated the correspondence of the bubbles and the DASS-21 subscales on the factor level. Specifically, the findings show that the depression bubble belongs to the same factor as the seven DASS-21 depression symptom items; the anxiety bubble belongs to the same factor as the seven DASS-21 anxiety symptom items; and the stress bubble belongs to the same factor as the seven DASS-21 stress symptom items. Furthermore, the AVEs provided first evidence for an adequate convergent validity of the three bubbles. Also, the analysis of the reliability of the bubbles in relationship with the DASS-21 subscales was adequate supporting their correspondence. Notably, the reliability of the DASS-21 subscales with and without the bubbles did not remarkably differ.

The very similar association pattern between both measures and the demographic variables contributed also to the confirmation of the correspondence. The significant negative association of the negative symptoms with age revealed higher levels of depression, anxiety and stress symptoms in younger people. This finding corresponds with previous research from Germany [44] and China [82]. It could at least partly be explained by the fact that the increase of mental health problems since the Covid-19 outbreak has particularly affected younger people [7, 12, 82–86].

In line with earlier research [49], the stress bubble was significantly negatively linked to gender revealing higher stress in female participants. However, the DASS-21 stress subscale and the other instruments were not significantly linked to gender. This finding was unexpected considering previous results on gender and the mental health variables [45, 48]. However, we found the non-significance for both the bubbles and the DASS-21 subscales. Thus, it seemed to be a characteristic of the investigated sample rather than a shortcoming of the bubbles.

All instruments were significantly negatively associated with social status revealing higher depression, anxiety and stress symptoms in people with a lower social status. This result corresponds with available literature [50, 51]. A low social status is often accompanied by low education and the lack of functional strategies to cope with daily hassles [52]. A permanent experience of stressful events in everyday life without the possibility of an adequate coping can negatively impact mental health and result in high levels of depression, anxiety and stress symptoms [87, 88].

Furthermore, the very similar association pattern of the bubbles and the DASS-21 subscales with variables of negative and positive mental health–as shown by the correlation and regression analyses–confirmed their correspondence and provided evidence for the convergent validity of the bubbles. In line with previous results [21, 43, 89], we found a significant positive association between depression, anxiety and stress symptoms–assessed by the bubbles and by the DASS-21 subscales. Notably, the three negative symptoms can enhance the level of each other [43].

PMH that represents the positive dimension of mental is an important protective factor against mental disorders and suicide-related outcomes [90, 91]. Persons with a high level of PMH are characterized by self-efficacy, resilience and the ability to cope with stressful experiences adequately [92]. In line with available literature [93, 94], the bubbles and the DASS-21 subscales were significantly negatively associated with PMH in the current study.

The predictive power of the bubbles and the DASS-21 subscales showed a similar result pattern. Thus, our findings revealed a high correspondence between the bubbles and the DASS-21 subscales and a good convergent validity of the three bubbles. Notably, their association with the DASS-21 subscales was weaker than the relationship between the subscales. However, the association of the bubbles and the DASS-21 subscales with PMH was similarly strong.

To sum up, the results of Study 1 provide initial evidence that the three bubbles are valid instruments for a rapid screening of the level of depression, anxiety and stress symptoms. Their correlations and predictive power are very similar to those of the 21 items of the DASS-21. Against this framework, we can assume that the use of the bubbles could be of great advantage due to their time- and cost-efficiency especially in large-scale representative studies and longitudinal studies.

## Study 2

### Methods

#### Procedure and participants

Overall, 1,066 persons started the survey and 172 (16.1%) dropped out. Thus, the sample of Study 2 consisted of 894 participants from Germany (see Table 1 for demographic variables derived from the present sample). The dataset used in the present study is available in S2 Dataset.

#### Measures

*Same instruments as in Study 1*. DASS-21 for the assessment of depression (current scale reliability of depression subscale: *α* = .922), anxiety (current scale reliability of anxiety subscale: *α* = .892) and stress symptoms (current scale reliability of stress subscale: *α* = .911); depression bubble, anxiety bubble, and stress bubble; PMH-Scale for the assessment of PMH (current scale reliability: *α* = .927).

*Further instruments*. ***Sense of control*.** Following Niemeyer, Bieda [52], we assessed sense of control by the two items “Do you experience important areas of your life (i.e., work, free-time, family, etc.) to be uncontrollable, meaning that you cannot, or barely can, influence them?” and “Do you experience these important areas of your life as unpredictable or inscrutable?”. The two items are rated on a 5-point Likert-type scale (0 = *not at all*, 4 = *very strong*). Higher sum scores indicate lower level of sense of control. The total sum score can range from 0 to 8. Current scale reliability is *α* = .865.

*Life satisfaction*. The unidimensional Satisfaction with Life Scale assessed life satisfaction (SWLS; original version: [95]; German version: [96]). This unidimensional instrument includes five items that are rated on a 7-point Likert-type scale (e.g., “In most ways, my life is close to my ideal.”; 1 = *strongly disagree*, 7 = *strongly agree*). The higher the sum score, the higher the level of life satisfaction. The total sum score can range from five to 35. Current scale reliability is *α* = .917.

*Perceived social support*. We assessed anticipated or perceived support received from the social network by the brief form of the Social Support Questionnaire (F-SozU K-6; original German language version: [66]) that is a unidimensional measure of social support. It consists of six items (e.g., “I experience a lot of understanding and security from others.”) that are rated on a 5-point Likert-type scale (1 = *not true*, 5 = *true*). Higher sum scores indicate higher levels of social support. The total sum score can range from 5 to 30. Current scale reliability is *α* = .901.

#### Statistical analyses

Statistical analyses were conducted using SPSS version 28 [71] and the open statistical software jamovi (version 2.3.26.0; www.jamovi.org). All investigated psychological variables were close to normally distributed (indicated by analyses of skewness, < 2.00, and kurtosis, < 7.00 [72]; see Table 4). An EFA and a CFA should not be calculated with the same sample [76, 77]. Therefore, we used the sample of Study 2 to replicate the unidimensional factor structure of the three DASS-21 subscales including the corresponding bubbles by the calculation of three CFAs. Again, we made the decision to calculate a CFA for each construct separately instead of a CFA that includes all three constructs (that are 24 items: 21 DASS items and the three bubbles) based on previous research that emphasized the need to consider depression, anxiety, and stress symptoms as separate entities [73, 74]. Thus, each CFA included eight items (seven items of a DASS-21 subscale and the corresponding bubble). Because of the sample size sensitivity of the chi-square test [97], we took further fit indices into consideration [98]: The comparative fit index (CFI), the root mean square error of approximation (RMSEA), and the standardized root mean residual (SRMR). Considering the cut-off criteria, values > .90 indicate a good fit for the CFI [99], values ≤ .08 indicate a reasonable fit for the RSMEA and values ≤ .05 reveal a good RSMEA fit [100], and values < .08 indicate a good fit for the SRMR [101]. We also examined the factor loadings of the CFAs, with a minimum for factor loading set at .400 [102]. Next, to assess the reliability of the bubbles, we calculated their internal consistency (Cronbach’s *α*) in relationship with the DASS-21 subscales. This allowed further conclusions on the correspondence between the bubbles and the DASS-21 subscales. Then, we focused again on the associations of the bubbles to replicate the findings of Study 1 and to extend them. We assessed the bubbles’ association with age, gender and social status by Pearson’s zero-order bivariate correlations and Spearman’s rank order correlations [79]. To gain further information about the convergent validity of the bubbles, we assessed their association with the DASS-21 subscales and sense of control (negatively coded), as well as the PMH-Scale, SWLS and F-SozU K-6 by the calculation of Pearson’s zero-order bivariate correlations. We also compared the associations of each bubble with the associations of the corresponding DASS-21 subscale (bubbles vs. DASS-21 subscale, effect size Cohen’s q [80]). Linear regression analyses that included, respectively, one of the bubbles as a predictor and, respectively, a DASS-21 subscale, sense of control, PMH-Scale, SWLS, or F-SozU K-6 as the outcome replicated the potential predictive power of the bubbles. Then, the regression analyses were calculated with the DASS-21 subscales, respectively, as a predictor. This step allowed a further comparison of both measures.

### Results

#### Factor structure of the bubbles in relationship with the DASS-21 subscales: Confirmatory Factor Analyses (CFAs)

Table 7 shows the results of the three CFAs. The CFA that included the depression bubble and the DASS-21 depression subscale in a one-factor structure resulted in a significant chi-square value. The CFI indicated a good fit, the RMSEA indicated a reasonable fit, and the SRMR indicated a good fit. The factor loadings ranged between .594 (depression bubble) and .773 (Item 3 of the DASS-21 depression subscale) (see Table 7).

**Table 7:** Fit indices and factor loadings of the confirmatory factor analyses (Study 2).

Confirmatory Factor Analyses	Factor Loading	*χ*^2^, *p*	CFI	RSMEA [90% CI]	SRMR
*1*.*CFA*: *Depression Symptoms*		128.0, < .001	.977	.078 [.065, .091]	.022
DASS-21: Depression Subscale Item 1	.635				
DASS-21: Depression Subscale Item 2	.621				
DASS-21: Depression Subscale Item 3	.773				
DASS-21: Depression Subscale Item 4	.765				
DASS-21: Depression Subscale Item 5	.754				
DASS-21: Depression Subscale Item 6	.757				
DASS-21: Depression Subscale Item 7	.715				
Depression Bubble	.594				
*2*. *CFA*: *Anxiety Symptoms*		136.0, < .001	.969	.081[.068, .094]	.028
DASS-21: Anxiety Subscale Item 1	.568				
DASS-21: Anxiety Subscale Item 2	.583				
DASS-21: Anxiety Subscale Item 3	.582				
DASS-21: Anxiety Subscale Item 4	.700				
DASS-21: Anxiety Subscale Item 5	.569				
DASS-21: Anxiety Subscale Item 6	.664				
DASS-21: Anxiety Subscale Item 7	.705				
Anxiety Bubble	.538				
*3*. *CFA*: *Stress Symptoms*		139.0, < .001	.971	.082 [.069, .095]	.028
DASS-21: Stress Subscale Item 1	.592				
DASS-21: Stress Subscale Item 2	.650				
DASS-21: Stress Subscale Item 3	.682				
DASS-21: Stress Subscale Item 4	.733				
DASS-21: Stress Subscale Item 5	.785				
DASS-21: Stress Subscale Item 6	.634				
DASS-21: Stress Subscale Item 7	.740				
Stress Bubble	.546				

*Notes*. *N* = 894; CFA = Confirmatory Factor Analysis; DASS-21 = Depression Anxiety Stress Scales 21; *χ*^2^ = Chi-Square Test; CFI = Comparative Fit Index; RMSEA = Root Mean Square Error of Approximation; CI = Confidence Interval; SRMR = Standardized Root Mean Residual; degrees of freedom (df) of chi-square tests = 20; overall three confirmatory factor analyses were calculated, each of them included eight items (= seven items of the DASS-21 subscale and the corresponding bubble).

The CFA that included the anxiety bubble and the DASS-21 anxiety subscale in a one-factor structure resulted in a significant chi-square value. The CFI indicated a good fit, the RMSEA indicated a reasonable fit, and the SRMR indicated a good fit. The factor loadings ranged between .538 (anxiety bubble) and .705 (Item 7 of the DASS-21 anxiety subscale) (see Table 7). The CFA that included the stress bubble and the DASS-21 stress subscale in a one-factor structure resulted in a significant chi-square value. The CFI indicated a good fit, the RMSEA indicated a reasonable fit, and the SRMR indicated a good fit. The factor loadings ranged between .546 (stress bubble) and .785 (Item 5 of the DASS-21 stress subscale) (see Table 7).

#### Association pattern of the bubbles and convergent validity

Table 4 shows the descriptive statistics of the bubbles and the other assessed variables (that are means, standard deviations, minimum, maximum).

*Correlations with demographic variables*. The three bubbles and the DASS-21 subscales were significantly negatively correlated with age and social status (see Table 5). The correlations did not significantly differ between the bubble and the subscale for depression (bubble vs. DASS-21 subscale: age: q = .031, social status: q = .032; both: no effect), anxiety (bubble vs. DASS-21 subscale: age: q = .074, social status: q = .036; both: no effect), and stress (bubble vs. DASS-21 subscale: age: q = .096, social status: q = .019; both: no effect). The stress bubble was significantly negatively correlated with gender. The other measures were not significantly correlated with gender (see Table 5). There were no significant differences between the correlations of the bubbles and the subscales (bubble vs. DASS-21 subscale: depression: q = .031, anxiety: q = .009, stress: q = .029; all: no effect).

*Convergent validity*. There was a significant positive correlation between depression, anxiety and stress symptoms–assessed by the bubbles and by the DASS-21 subscales. The depression bubble was significantly positively correlated with the anxiety bubble, *r* = .489, *p* < .001, and the stress bubble, *r* = .571, *p* < .001. The anxiety bubble was significantly positively correlated with the stress bubble, *r* = .387, *p* < .001.

Each bubble was significantly positively correlated with the corresponding DASS-21 subscale (see Table 5). Furthermore, both depression measures were significantly positively correlated with DASS-21 anxiety symptoms (DASS-21 > bubble: q = .435, medium effect) and DASS-21 stress symptoms (DASS-21 > bubble: q = .676, large effect). Both anxiety measures were significantly positively correlated with DASS-21 depression symptoms (DASS-21 > bubble: q = .604, large effect) and DASS-21 stress symptoms (DASS-21 > bubble: q = .680, large effect). Both stress measures were significantly positively correlated with DASS-21 depression symptoms (DASS-21 > bubble: q = .578, large effect) and DASS-21 anxiety symptoms (DASS-21 > bubble: q = .412, medium effect) (see Table 5). Furthermore, both depression measures (DASS-21 > bubble: q = .253, small effect), both anxiety measures (DASS-21 > bubble: q = .201, small effect), and both stress measures (DASS-21 > bubble: q = .352, medium effect) were significantly positively correlated with a low level of sense of control (see Table 5).

Considering the variables that represent the positive dimension of mental health, the bubbles and the DASS-21 subscales were significantly negatively correlated with PMH (depression: q = .042; anxiety: q = .001; stress: q = .054, all: no effect) (see Table 5). Moreover, all measures were significantly negatively correlated with life satisfaction (depression: q = .031; anxiety: q = .015; stress: q = .029, all: no effect) and social support (depression: q = .005; anxiety: q = 0; stress: q = .014, all: no effect) (see Table 5).

#### Predictive power of the bubbles

As shown in Table 6, all investigated regression models were significant revealing that the bubbles could predict symptoms of depression, anxiety, and stress, sense of control, PMH, life satisfaction, and social support. Again, the association of the bubbles with the negative variables was weaker than the association of the DASS-21 subscales. However, the association between the bubbles and the positive variables did not remarkably differ from the association of the DASS-21 subscales (see Table 6).

### Discussion

Study 2 aimed to replicate and to extend the findings of Study 1 on the validity of the three new developed bubbles. The CFAs confirmed the results of the EFAs in Study 1. Thus, the one-factor models fit the data well for depression, anxiety, and stress. This again emphasized the correspondence of the bubbles and the DASS-21 subscale on the factor level. The correlation analyses and the regression analyses replicated the result pattern of Study 1. In line with available literature [44, 51] and the findings of Study 1, the three bubbles and the DASS-21 subscales were significantly negatively associated with age and social status revealing higher depression, anxiety and stress symptoms in younger persons and in persons with a lower social status. Also in line with Study 1, the stress bubble was significantly negatively associated with gender revealing a higher stress level in female participants. Thus, the bubbles and the DASS-21 subscales showed again very similar correlations with demographic variables which emphasizes their correspondence.

In line with earlier research [21] and Study 1, the bubbles and the DASS-21 subscales were significantly positively associated which provided further support for their correspondence. Also, high levels of both depression measures, both anxiety measures and both stress measures were accompanied by a low level of sense of control. This finding corresponds with available literature [60]. A loss of control in important areas of everyday life is a significant characteristic of the negative symptoms [103]. This provides further support of the bubbles’ convergent validity.

As a further replication of Study 1 findings and in line with available literature [60], the bubbles and the DASS-21 subscales were significantly negatively linked to PMH. Notably, life satisfaction and social support are often considered as further factors of the positive dimension of mental health [62]. While they often exhibit a similar association pattern to PMH, it is important to note that the associations are not always consistent. Therefore, it has been recommended to investigate all of them to assess a wide-ranging spectrum of the positive dimension [62, 104, 105]. Against this background, we added the variables life satisfaction and social support in the present investigation of the validity of the bubbles. Notably, the three bubbles and the DASS-21 subscales were significantly negatively linked to life satisfaction and social support. This finding was in line with previous research [43, 56, 65].

Furthermore, in line with Study 1, the predictive power of the bubbles and the DASS-21 subscales showed a very similar pattern. Thus, the results replicated the correspondence of the three bubbles and the DASS-21 scales as well as the bubbles’ convergent validity. Again, their association with the negative variables (i.e., DASS-21 subscales, sense of control) was weaker than the associations of the DASS-21 subscales. However, the association of the bubbles and the subscales with the positive variables was similarly strong.

To sum up, the findings of Study 2 confirmed the evidence gained in Study 1 that the three bubbles are valid time- and cost-efficient instruments for a brief screening of the level of depression, anxiety and stress symptoms.

## General discussion

More than 970 million people around the globe suffer from mental health problems [1]. Depression and anxiety disorders belong to the most common [1]. This has a negative impact not only on the affected individuals but also on the worldwide economy [106, 107]. Following the World Health Organization (2), depression and anxiety cost the global economy about one trillion US dollars annually. Depression and anxiety are closely associated with stress symptoms [21, 108]. By an early identification of persons at risk for enhanced depression, anxiety and stress symptoms, we could prevent their further development and maintenance. This would alleviate the suffering of the affected persons and their families, and it could have positive effects on both local and global economies.

To achieve this aim, a brief and valid assessment of the negative symptoms is of great importance. It could help to identify persons at risk and to observe potential changes of the symptoms over time. Against this background, we developed and validated three ultra-short scales for the assessment of depression, anxiety and stress symptoms–the “bubbles” in two population representative studies.

Our results demonstrated that the three bubbles are valid instruments that due to their shortness could be practical and economical tools for an initial very brief screening of the level of the negative symptoms in German language samples. Notably, we replicated the findings of previous research that used longer instruments for the assessment of depression, anxiety and stress symptoms.

The findings of Study 1 showed that the bubbles fit the DASS-21 subscales on the factor level. Furthermore, the bubbles were sensitive enough to replicate the association pattern of the subscales with demographic variables, and with variables that belong to the negative and the positive dimension of mental health.

The findings of Study 2 further confirmed the correspondence between the bubbles and the DASS-21 subscales. Again, the bubbles replicated the association pattern of the subscales for demographics and for further positive and negative variables.

Notably both studies revealed that the associations between the bubbles and the representatives of the negative dimension of mental health were weaker than the associations of the DASS-21 subscales with those variables. However, there was no significant difference considering the associations of the bubbles and the DASS-21 subscales with representatives of the positive dimension of mental health. This finding is in line with earlier research that validated the Single-Item Self-Esteem Scale (SISE) [34] that is an single-item measure of self-esteem. The authors compared SISE with the 10-item Rosenberg Self-Esteem Scale (RSE-Scale) [109, 110]. The relationship between the depression symptoms and self-esteem assessed by the SISE was remarkably weaker than their association with self-esteem measured by the RSE-Scale. However, the SISE self-esteem was strongly associated with subjective happiness and perceived social support [34]. Therefore, it could be that the single-item scales are especially suitable for investigations of associations between the appropriate construct and the positive dimension of mental health. This, however, does not mean that they should not be used in investigations that focus on the negative dimension of mental health.

Thus, similar to other single-item scales (e.g., risk-taking [35], happiness [38]), the bubbles seem to be a valid and economic instrument for a brief screening of the level of depression, anxiety and stress symptoms. Due to their time- and cost-efficiency, the bubbles can be of specific benefit in large-scale surveys, in longitudinal studies in order not to overload participants, to prevent cognitive fatigue and, therefore, attention deficits and enhanced drop-outs, and when a brief screening, for example in mental health prevention programs or assessment centers, is required. Moreover, the bubbles can be of significant benefit in research using the experience sampling paradigm to assess for example the link between the negative symptoms and specific experiences in everyday life. Considering their close correspondence with the DASS-21 subscales, the bubbles could be also used in addition to the subscales for example in experimental research. Specifically, in a study that includes a two-week intervention, the DASS-21 subscales could be used in the baseline measurement and the post-measurement to assess the negative symptoms, and the bubbles could assess the negative symptoms in a brief daily screening during the intervention period. In addition, some research uses a paper-and-pencil format for data collection, followed by a transfer of the data into a digital format. This process typically requires a double-checking and is susceptible to imputation errors. The larger the number of items included in the data collection, the more time is required for the transfer and double-checking and the higher the risk for imputation errors [111]. The shortness of the bubbles can mitigate data processing time and errors in such studies. Moreover, it reduces the amount of paper required, contributing to sustainability and climate protection.

### Limitations and future research

The following limitations should be taken into account when interpreting our findings. First, the cross-sectional study design did not allow us to assess the test-retest reliability of the bubbles. The investigation of this form of reliability is important, as single-item scales could be prone to lower reliability than instruments that consist of several items [112, 113].

And we could not assess the time sensitivity of the bubbles–specifically, whether they can measure short- and longer-term changes of depression, anxiety, and stress symptoms. Therefore, we suggest future research to test the bubbles by a longitudinal study design. Second, in order not to overload the participants, we included only a limited number of variables for the examination of the validity of the bubbles and we focused on their convergent validity. Future validation studies of the bubbles should include further measures of depression, anxiety and stress symptoms. Moreover, they should include further negative variables to examine the convergent validity of the bubbles such as insomnia and suicide-related outcomes [114], as well as variables that are not or only very weakly related to the negative symptoms to assess the discriminant validity of the bubbles. Third, future research should examine the validity of the bubbles in clinical patients with different diagnoses. This would provide evidence on the applicability and effectiveness of the bubbles as a measure of negative mental health in various clinical populations. Furthermore, the present work is the initial step for the development and validation of the three bubbles. So far, they are not considered as sensitive instruments for a clinically relevant diagnosis of for example an anxiety disorder. Future studies are required to evaluate their diagnostic accuracy and to specify cut-off points/values for clinically relevant levels of depression, anxiety and stress symptoms. Therefore, investigations in clinical populations would also be of advantage. Hereby, we recommend to involve measures of diagnostic accuracy such as the Area under the Recover Operating Characteristic (AuROC) curve, sensitivity and specificity, positive and negative predictive values (PPV, NPV) [115, 116]. Fourth, we developed the bubbles by transforming the DASS-21 items into short phrases or single words. We cannot exclude the possibility that this transformation potentially impacted the content validity of the constructs we converted into bubbles. Against this background, we recommend future research to assess whether the content of the bubbles can be understood in the same way as that of the DASS-21 subscales. Focus groups that discuss the content of both instruments could be a supportive initiate step in doing so (see also [117]). Furthermore, to test the correspondence of the depression bubble with the DASS-21 depression subscale, of the anxiety bubble with the DASS-21 anxiety subscale, and of the stress bubble with the DASS-21 stress subscale on the factor level, we calculated three EFAs in Study 1 and did not focus on the 21 items of the DASS-21 as an overall concept. Therefore, the analysis of whether the single items of the DASS-21 and the single bubbles have a propensity to be associated with other factors or components of the instrument or not has been neglected. Future research is recommended to test the 21 items of the DASS-21 and the three bubbles in one EFA to further elaborate this issue. Fifth, the validity of the DASS-21 has been shown in various countries [27]. We tested the bubbles in German samples only. Further translations of the bubbles to other languages as German and their validations in other countries are desirable. Sixth, we developed the bubbles only for symptoms of negative mental health. Considering that mental health includes a positive dimension also, the development and validation of further bubbles for variables such as PMH and life satisfaction are desirable.

## Conclusion

To sum up, the depression bubble, the anxiety bubble, and the stress bubble revealed to be valid, cost- and time efficient instruments for a brief screening of the level of the negative symptoms. Their use in research and praxis could speed up the working processes and contribute to a rapid identification of persons at risk for enhanced levels of depression, anxiety and stress symptoms as well as related mental health problems. Future studies are required for the validation of the bubbles in clinical context and cross-national.

## Supporting information

S1 Dataset Dataset used for analyses in present study (Study 1). (XLSX)

S2 Dataset Dataset used for analyses in present study (Study 2). (XLSX)
